# Suicidal ideations among medical students: The role of anhedonia and type D personality

**DOI:** 10.1371/journal.pone.0217841

**Published:** 2019-06-21

**Authors:** Gwenolé Loas, Alice Solibieda, Marianne Rotsaert, Yvon Englert

**Affiliations:** Department of Psychiatry & Laboratory of Psychiatric Research (ULB 266), Cliniques universitaires de Bruxelles, Université Libre de Bruxelles (ULB), Bruxelles, Belgium; University of Toronto, CANADA

## Abstract

**Background:**

The relationships between hedonic deficits, type D personality and suicidal ideation were explored in a group of medical students.

**Methods:**

In a cross-sectional study, 382 medical students filled out several questionnaires measuring suicide risk, depression (using the Beck Depression Inventory, i.e. BDI), type D personality (using the type D personality scale-14, i.e. DS-14) and anhedonia (using the anhedonia subscale of the BDI, the Snaith Hamilton Pleasure Scale, the Anticipatory and Consummatory subscales of the Physical Anhedonia Scale).

**Results:**

State anhedonia and, in particular, recent change of state anhedonia and not trait anhedonia was significantly associated with suicidal ideation, specifically when depression was controlled for. Negative affectivity component of type D personality and anhedonia were independent predictors of suicidal ideation even when depression was controlled for. Loss of pleasure and not loss of interest was a significant predictor of suicidal ideation.

**Conclusions:**

Change of state anhedonia and its component of loss of pleasure measuring dissatisfaction in life could be a risk factor of suicidal ideation in medical students. Dissatisfaction, particularly in the medical course, could be a strong predictor of suicidal ideation in medical students.

## Introduction

In the world, among adolescents and young adults 8.5% of all deaths are explained by suicide. Suicide is the leading cause of death among youth (15–29 years) worldwide. Lifetime prevalence rates of suicide attempts among youth range from 3.1% to 8.8% [1] and the prevalence rates of suicidal ideation for this population range from 19.8% to 24%. Several risk factors of suicidal thoughts and behaviors have been identified using either cross-sectional studies or prospective studies.

In a recent meta-analysis the prevalence of suicidal ideation among medical students was 11.1% [2]. Many factors contribute to the development of suicidal ideation in this population. Among these factors, high rates of burnout, depression and certain personality traits (neuroticism, low self-esteem…) have been described [3].

Specific personality traits or personality disorders associated with suicidal ideation have rarely been studied in medical students.

Distressed or type D personality is characterized by high levels of negative affectivity and social inhibition [4]. Type D personality has been associated with the risk of suicidal ideation in the general population [5, 6] and in patients who suffer from major depressive disorder [7]. High prevalence of type D personality has been found in different samples of medical students or physicians with a range from 27.2% to 35% [8] but the associations between this personality type and suicidal ideations have, as yet, not been studied in physicians or medical students.

Cross-sectional and prospective findings have shown that anhedonia, the lowered ability to experience pleasure, is associated with suicidal thoughts and behaviors in adolescents [1]. Moreover, anhedonia is associated with short-term (within one year) suicide risk in major depressive disorders [9]. The relationship between anhedonia and suicidal ideations is the most reported and the most strongly supported relationship.

As observed in a recent meta-analysis [10], anhedonia has been shown to be associated with suicidal ideation in various samples including undergraduate students [11]. In this meta-analysis, the association between anhedonia and suicidal ideation remained significant even when depression or psychiatric disorders were controlled for. More recently, a study reported that dental and medical training courses tend to promote anhedonia [12]. However, the relationship between anhedonia and suicidal ideation has not yet been explored in medical students.

Several authors have suggested taking into account the heterogeneity of anhedonia when examining the relationship with suicidal ideation. One hypothesis is that state- anhedonia and not trait-anhedonia was associated with suicidal ideations [13] and another hypothesis is that recent change of state anhedonia and not trait anhedonia is associated with suicidal ideations [14]. According to Winer et al [14] the available tools to measure state anhedonia, such as the Snaith Hamilton Pleasure Scale (SHAPS, [15]), do not allow a clear distinction with trait-anhedonia. Several studies [14, 16, 17] measuring state anhedonia with the SHAPS, recent change of state anhedonia with the Specific Loss of Interest and Pleasure Scale (SLIPS, [18]) or the anhedonia subscale of the Beck Depression Inventory (BDI-II), and trait-anhedonia with the Temporal Experiences of Pleasure Scale (TEPS) [19] reported that state- anhedonia and recent change of state anhedonia were associated with suicidal ideations. Trait-anhedonia, however, was not. Regarding recent change of state anhedonia, several studies using psychiatric subjects or university students [11, 14, 16] found that the social component of anhedonia, as measured by item number 12 (i.e. loss of interest) of the BDI or the SLIPS, relates specifically to suicidal ideations even when depressive symptoms are controlled for.

Taking into account physicians’ high suicide risk and the potential link between physicians’ job dissatisfaction and suicide risk [20], the relationship between suicidal ideations and anhedonia has been explored in a recently published study [21]. More precisely, the study explored the association between recent suicidal ideation and recent change in state-anhedonia whilst also taking into account the potential effect of depression. However, the separate effect of the social component of anhedonia or the lack of pleasure (thus also measuring dissatisfaction) was examined too. Dissatisfaction is the state or attitude of not being satisfied with a feeling of displeasure or disappointment. It comprises two components: lack of pleasure and negative affect with displeasure.

In a recent cross-sectional study in 557 physicians [21], significant relationships were found between recent change of state-anhedonia as measured by the anhedonia subscale of the BDI and suicidal ideation even when significant variables were taken into account and in particular depressive symptoms. Among the different components of anhedonia, only loss of satisfaction (item 4 of the BDI) and not the loss of interest in other people (item 12) showed a significant relationship with suicidal ideation. Thus, contrary to what had been reported in university students or psychiatric subjects, physicians’ suicidal ideations seemed especially associated with loss of pleasure and was most certainly not limited to the loss of interest in people.

Anhedonia and type D personality are both associated with suicidal ideation and have both recently been studied in medical students [22]. In a sample of 204 medical students, significant relationships have been reported between the social inhibition component of the type D personality and anhedonia as rated by the Snaith Hamilton Pleasure Scale even when the depressive level was controlled for using partial correlations. The relationships between anhedonia and type D personality, however, could not be explained solely by looking at their respective relationships with depression, thus suggesting that social inhibition could be the common factor between the two dimensions. Social inhibition could also be related to suicidal ideation independently of depression.

To the best of our knowledge, the relationships between anhedonia, type D personality and suicidal ideation have, as yet, not been examined in medical students. Therefore, the purpose of the present study was to establish these relationships. To this effect, several hypotheses have been examined.

Firstly, we explored if recent change of state anhedonia and not trait anhedonia were associated with suicidal ideation and if this association is independent of depression. This first hypothesis is a replication of previous studies with medical students.

Secondly, we examined if anhedonia was an independent predictor of suicidal ideation comparatively with type D personality when taking into account the potential effect of depression. One possibility is that only social inhibition is a significant predictor of suicidal ideations. Another possibility is that anhedonia and social inhibition or negative affectivity are independent predictors of suicidal ideations.

Thirdly, we examined if suicidal ideations were associated with loss of interest or loss of pleasure. If suicidal ideations in medical students were associated with the component of loss of interest of anhedonia then the pattern of correlation would be similar to the one observed in university students. If suicidal ideations in medical students were associated with loss of pleasure, measuring dissatisfaction, then the pattern of correlation would be similar to the one observed in physicians.

The main interest of testing these hypotheses was to identify the relevant predictors (anhedonia versus type D personality, loss of interest versus loss of pleasure) of suicidal ideations in medical students thus making it possible to set up specific prevention programs similar to those used with physicians.

## Methods

### Participants

In order to examine the working hypotheses, 382 medical students (155 men, 227 women) with a mean age of 22.82 years (sd = 1.96) were recruited. Subjects for this study came from two separate groups of medical students. The first group had been used in a previous study examining the relationships between anhedonia and type D personality [22]. This study which was approved by the Ethics Committee of the Hôpital Erasme involved 204 (102 men, 102 women) undergraduate medical students (from all academic years) recruited at the Université Libre de Bruxelles (ULB) and the Université of Mons (UMons). The aim of the study was explained in an information document and all participants gave their signed written informed consent.

The second group was made up of 178 (53 men, 125 women) undergraduate medical students (63 in 2^nd^ year Bachelor’s degree, 115 in 3^rd^ year Master’s degree) and was recruited at the ULB. The study was approved by the Ethics Committee of the Hôpital Erasme. The participation was voluntary and act of logging on the online data collection system was used as informed consent.

To take part in the study, participants had to be French-speaking students, aged 18 years and older. There were no exclusion criteria. For the first group the questionnaires were completed in a classroom setting and for the second group the questionnaires were administered via an online data collection system.

### Measures (see Table 1)

We used self-rating scales exclusively.

**Table 1:** Psychometric characteristics of the samples.

	N	Mean	SD	Range
**PAS-ANT**	204	2.67	1.45	0–7
	102 Females	2.58	1.42	
	102 Males	2.76	1.48	
**PAS-CONS**	204	5.81	2.94	0–14
	102 Females	5.45	2.84	
	102 Males	6.17	3.00	
**SHAPS**	204	23.04	4.43	14–36
	102 Females	22.18	4.32	
	102 Males	23.91	4.40	
**Anhedonia—BDI**	382	1.27	1.54	0–8
	227 Females	1.47	1.61	
	155 Males	0.99	1.37	
**LP**	382	0.43	0.62	0–3
	227 Females	0.48	0.65	
	155 Males	0.36	0.57	
**LI**	382	0.51	0.70	0–3
	227 Females	0.61	0.75	
	155 Males	0.37	0.60	
**LIS**	382	0.33	0.69	0–3
	227 Females	0.38	0.72	
	155 Males	0.26	0.64	
**NA**	382	10.8	5.98	0–26
	227 Females	11.47	5.69	
	155 Males	9.81	6.27	
**SI**	382	12.29	5.43	0–26
	227 Females	12.45	5.23	
	155 Males	12.05	5.71	
**CA-BDI**	382	3.57	3.34	0–17
	227 Females	3.84	3.57	
	155 Males	3.16	2.94	

(PAS-ANT: Anticipatory subscale of the physical anhedonia scale; PAS-CONS: Consummatory subscale of the physical anhedonia scale; SHAPS: Snaith-Hamilton pleasure scale; BDI: Beck Depression Inventory; LP: Loss of pleasure; LI: Loss of Interest; LIS: Loss of Interest in Sexual activity; NA: Negative affectivity subcale of the type D personnality scale; SI: Social inhibition subscale of the type D personnality scale; CA-BDI: Cognitive-Affective subscale of the BDI)

#### Anhedonia

Three measures of anhedonia were used rating trait-anhedonia, state-anhedonia and recent change of state-anhedonia.

Trait-anhedonia was rated using the Anticipatory and Consummatory subscales of the Physical Anhedonia Scale [23]. The PAS anticipatory (PAS-ANT) and PAS consummatory (PAS-CONS) scales have been designed by taking into account correlations between the items of the revised Physical Anhedonia Scale (PAS) and the TEPS-CONS or TEPS-ANT scales. Ten items for the PAS-ANT and 16 items for the PAS-CONS subscales were selected. These two subscales presented satisfactory psychometric properties [23]. Total scores for the PAS-ANT and PAS-CONS ranged from 0 to 10 and 0 to 16, respectively, and were related to the level of trait-anhedonia. Trait-anhedonia was assessed only in the first group of medical students.

State-anhedonia was rated using the SHAPS [15] that evaluates an individual’s state of pleasure experienced in recent days (for example: “I have enjoyed being with my family or close friends”). The scale consists of 14 items rated on a 4-point Likert scale from 1 (“I strongly agree”) to 4 (“I strongly disagree”). The total score ranges from 14 to 56. The level of pleasure is inversely related to the score of the scale. The French version of the scale has satisfactory psychometric properties [24] with a value of .8 for the Cronbach alpha and a test-retest r of .56 over a one-month period. State-anhedonia was assessed only in the first group of medical students.

Recent change of anhedonia was evaluated using the anhedonia subscale (ANH-BDI) of the Beck Depression Inventory-II (BDI-II) [25] that contains three items (Item 4 or Loss of Pleasure (LP) ‘I can’t get any pleasure from the things I used to enjoy’; item 12 or Loss of Interest (LI) ‘It’s hard to get interested in anything’ and item 21 or Loss of Interest in Sex (LIS) ‘I have lost interest in sex completely’). In the original version of the BDI item 4 was labeled “lack of satisfaction” showing notably that lack of satisfaction and loss of pleasure are two overlapping concepts. This subscale has been validated [26] in several samples of psychiatric subjects with Cronbach alpha ranging from 0.57 to 0.73. Moreover, one confirmatory factorial analysis found that the two-factor model of the BDI-II (anhedonia-subscale and BDI with the remaining 18 items) had higher adequacy indices than the one-factor model [25].

#### Type D personality

The Type D personality scale (DS-14) consists of two seven-item subscales. It has been specifically developed to assess negative affectivity (NA) and social inhibition (SI) [4]. Each item is rated from 0 to 4 on a 5-point Likert scale. The total score for each subscale ranges from 0 to 28. Satisfactory validity and reliability have been reported. NA and SI subscales had high internal consistencies and temporal stabilities (the Cronbach alpha coefficients were .88 and .86, respectively, and the test-retest r was .72 and .82 over a 3-month period) [4]. The French version of the scale has satisfactory psychometric properties [27].

#### Depression

Depression was rated using the revised version of the Beck Depression Inventory (BDI) [25]. The BDI uses statements that best describe how the individual has felt during the previous two weeks. The French version of the BDI-II has satisfactory psychometric properties [28]. The total score ranges from 0 to 63 and higher total scores indicate more severe depressive symptoms. For the present study, cognitive-affective symptoms of depression (CA-BDI) were assessed using the items relating to past failure, guilty feelings, punishment feelings, self-dislike, self-criticalness and worthlessness. This subscale of the BDI has been used previously by Winer et al [14].

Suicidal ideation (SID) was rated using the “Suicidal thoughts and wishes” item of the BDI-II in which a 0 is “I don't have any thoughts of killing myself” and 3 is “I would like to kill myself if I had the chance”.

### Statistical analyses

Firstly, bivariate statistical analyses were performed using the dependent variable (Suicidal ideation, SID), the independent variables (ANH-BDI, LP-BDI, LI-BDI, LIS-BDI, PAS-ANT, PAS-CONS, SHAPS, NA, SI, CA-BDI) and two covariates (gender, age). Pearson’s correlation and Student’s t test were done. The level of significance was p < 0.05.

Secondly, multiple regressions were performed using recent change of suicidal ideation as dependent variables, covariates and independent variables which had been found to be significant in the bivariate analyses. Ridge regressions were used to take into account the multicollinearity between the variables.

## Results (see Table 2)

### First hypothesis

The first hypothesis, using the first sample (N = 204), tested if recent change of state anhedonia (anhedonia subscale of the BDI, ANH-BDI) or state anhedonia (Snaith Hamilton Pleasure Scale, SHAPS) and not trait anhedonia (Anticipatory or Consummatory subscale of the Physical Anhedonia Scale, PAS-ANT or PAS-CONS) were associated with suicidal ideation.

**Table 2:** Multiple linear ridge regression analyses predicting suicidal ideation.

Predictors				
	*ß*	*Student’s t test*	*p*	*% Variance*
*First hypothesis (N = 204)*				
SHAPS	0.047	0.76	0.44	
PAS-ANT	0.013	0.21	0.83	
Anhedonia- BDI	**0.22**	**3.08**	**0.0023**	**4.26**
Cognitive/Affective-BDI	**0.30**	**4.34**	**0.0001**	**19.89**
*Second hypothesis (N = 382)*				
NA	**0.163**	**3.14**	**0.002**	**2.61**
SI	0.076	1.73	0.084	
Anhedonia-BDI	**0.224**	**4.54**	**0.0001**	**5.96**
Cognitive/Affective-BDI	**0.22**	**4.19**	**0.0001**	**21.73**
*Third hypothesis (N = 382)*				
LP	**0.246**	**5**	**0.0001**	**6.71**
LI	0.053	1.09	0.27	
LIS	**0.09**	**2.03**	**0.042**	**1.09**
Cognitive/Affective-BDI	**0.28**	**5.69**	**0.0001**	**21.73**

(SHAPS, Snaith Hamilton Pleasure Scale; PAS-ANT, anticipatory subscale of the Physical Anhedonia Scale; ANH-BDI, anhedonia subscale of the Beck Depression Inventory-II (BDI); NA, negative affectivity subscale, SI, social inhibition subscale of the Type D personality scale; LP, loss of pleasure; LI, loss of interest; LIS, loss of interest in sex, ß, standardized regression coefficient, in bold face p < 0.05).

#### Bivariate analyses

There was no significant association between age or gender and suicidal ideation.

ANH-BDI, SHAPS, PAS-ANT, CA-BDI presented significant correlations with suicidal ideation. PAS-CONS did not correlate with suicidal ideation. Thus, only trait-consummatory anhedonia was not significantly associated with suicidal ideations.

#### Multivariate analysis

Linear ridge regression was performed using suicidal ideation as the dependent variable and SHAPS, PAS-ANT, ANH-BDI and CA-BDI as four independent variables. The global test was significant (F (4, 199) = 16.09, p < 0.0001). Two predictors were significant, CA-BDI (t (199) = 4.34, p < 0.0001) and ANH-BDI (t (199) = 3.08, p < 0.002) explaining 19.89 and 4.26%, respectively, of the variance. Of the four anhedonia scales that were significantly correlated with suicidal ideations in bivariate analyses, only ANH-BDI (rating recent change of state-anhedonia) remained significantly correlated with suicidal ideations when the other anhedonia scales and the cognitive-affective component of depression were controlled for. Thus, the effect of recent change of state-anhedonia on suicidal ideations was independent of depression.

### Second hypothesis

The second hypothesis, using the entire sample (N = 382), examined if anhedonia and type D personality were independent predictors of suicidal ideation.

#### Bivariate analyses

There was no significant association between age or gender and suicidal ideation.

ANH-BDI, SI, NA, CA-BDI had significant correlations with suicidal ideation.

#### Multivariate analysis

Linear ridge regression was performed using suicidal ideation as the dependent variable and NA, SI, ANH-BDI and CA-BDI as four independent variables. The global test was significant (F (4, 377) = 42.05, p < 0.0001). Three predictors were significant, CA-BDI (t (377) = 4.19, p < 0.0001), ANH-BDI (t (377) = 4.54, p < 0.0001) and NA (t (377) = 3.14, p < 0.002) explaining 21.73%, 5.96% and 2.61%, respectively, of the variance. Recent change of state-anhedonia and the negative affect component of the type D personality were independent predictors of suicidal ideations even when the cognitive-affective component of depression was controlled for.

### Third hypothesis

The third hypothesis examined if suicidal ideations were associated with loss of interest in people or loss of pleasure.

#### Bivariate analyses

All three items (Loss of Pleasure, Loss of Interest, Loss of Interest in Sex) of the ANH-BDI correlated significantly with suicidal ideation.

#### Multivariate analysis

Linear ridge regression was performed using suicidal ideation as the dependent variable and LP, LI, LIS and CA-BDI as four independent variables. The global test was significant (F (4, 377) = 39.58, p < 0.0001). Three predictors were significant, CA-BDI (t (375) = 5.68, p < 0.0001), LP (t (377) = 5, p < 0.0001) and LIS (t (377) = 2.03, p < 0.043) explaining 21.73%, 6.71% and 1.09%, respectively, of the variance. Only the components “loss of pleasure” and “loss of interest in sex” were independent predictors of suicidal ideations even when the cognitive-affective component of depression was controlled for.

## Discussion

The first hypothesis explored if the various hedonic deficits were associated with suicidal ideation and whether these significant associations were still found when potential effect of the depressive symptomatology was taken into account.

When depression was controlled for using the cognitive-affective dimension of the depressive symptomatology using the CA-BDI and when the effects of the different hedonic deficits were studied together, then only recent change of anhedonia, using the anhedonia subscale of the BDI, was a significant predictor of suicidal ideation. A recent meta-analysis [10] compared 657 subjects with current suicidal ideation and 6690 subjects without current suicidal ideation. Higher levels of anhedonia were found in the group with current suicidal ideation compared to the group without suicidal ideation with a medium effect size (standardized mean difference = 0.57, z = 5.43, P <0. 001, 95% confidence interval, CI = 0.37–0.79). The association did not change when controlling for depression and psychiatric disorders. In this study the type of anhedonia was not taken into account.

So far, only two studies have explored the relationships between trait and state anhedonia and suicidal ideations.

In 122 patients hospitalized for mood and anxious disorders [17], suicidal ideation (as rated by the “Suicidal thoughts and wishes” item of the BDI-II), was significantly correlated with two anhedonia scales: the ANH-BDI (Spearman ρ = 0.45, p < 0.001) and the PAS-ANT (ρ = 0.21, p < 0.05). However, no significant correlations were reported between suicidal ideation and PAS-CONS (ρ = 0.12), TEPS-ANT (ρ = - 0.1) or TEPS-CONS (ρ = - 0.15).

The second study [29] used a sample of 395 psychiatric outpatients who filled out the Columbia suicide severity rating scale, the Beck scale for suicidal ideation, the BDI-II, the Spielberger state-trait anxiety inventory and the modified SHAPS. At a 1-month follow-up, 289 of the original study subjects filled out the various measures of suicidal ideations. Based on their responses to the Snaith Hamilton Pleasure Scale at initial assessment, patients had been classified as acutely anhedonic, chronically anhedonic and non-anhedonic. Acute anhedonia at initial assessment and at follow-up were found to be associated with higher severity of suicidal ideation compared to the non-anhedonic group even when symptoms of anxiety and depression were controlled for. There was no difference in severity of suicidal ideation between non-anhedonic and chronically anhedonic groups at either point in time.

One study [30] examined the relationship between suicidal ideations and state anhedonia rated by the SHAPS and recent change of anhedonia rated by the ANH-BDI in 100 treatment-resistant patients presenting mood disorders and treated by ketamine. The scale for suicide ideation and the Hamilton Depression rating Scale were used. The relationship between anhedonia and suicidal ideation was examined before and after ketamine administration. The SHAPS and not the anhedonia subscale of the BDI was significantly associated with suicidal ideation before and after ketamine administration even when the depressive symptoms were controlled for.

Thus, state-anhedonia with or without recent change and not trait anhedonia is related to suicidal ideation when depression or anxiety are controlled for.

The second hypothesis explored if anhedonia and type D personality were independent predictors of suicidal ideation.

A previous study [22], using the first sample of the present study, observed significant association between anhedonia, rated by the SHAPS, and the Social Inhibition subscale of the type D rating scale. The correlation remained significant when depression was controlled for using the BDI-II scores. In the present study, anhedonia as rated by the ANH-BDI and Negative affectivity subscale of the type D personality was an independent predictor of suicidal ideation when depression was controlled for. Interestingly, this could be indicative that anhedonia and negative affectivity could represent the two components of dissatisfaction. Medical students’ dissatisfaction with their courses could be the equivalent of physicians’ job dissatisfaction.

So far, there has been no study that has explored the relationship between anhedonia, type D personality and suicidal ideations. Several programs have been developed for medical students with the aim to improve their well-being, to detect untreated and potentially suicidal students anonymously and to increase mental health services utilization [3, 31]. Taking into account the results of the present study it could potentially be helpful to incorporate the anhedonia subscale of the BDI-II and the type D personality scale in future suicide prevention programs.

The third hypothesis examined if suicidal ideations in medical students were associated with loss of interest or loss of pleasure.

Previous studies focusing on psychiatric subjects or university students have shown that, of all the components of the anhedonia scales, the loss of interest, particularly in people, was the most predictive of suicidal ideation.

The relationships between anhedonia (using the anhedonia subscale of the BDI-II) and suicidal ideations (using the Suicidal thoughts and wishes item of the BDI-II) have been studied in a sample of 1529 psychiatric subjects [14]. There were two assessments, one at intake and another one 6 weeks later. Regression analyses reported that the anhedonia subscale was a strong predictor of suicidal ideation at baseline and termination, even when cognitive/affective symptoms of depression were controlled for. The authors then examined the relationships between each item of the anhedonia subscale of the BDI and suicidal ideation at the two different points in time. All four anhedonia items were entered together in the regression analyses. Firstly, the authors examined if the anhedonia items at baseline predicted suicidal ideation at baseline and, secondly, they examined if the anhedonia items at baseline predicted suicidal ideation at termination. Of the four anhedonia items, only loss of interest significantly predicted suicidal ideation both at baseline and at termination. The authors concluded that loss of interest was the strongest predictor of suicidal ideation.

In a second study, the same team [11] examined the association between anhedonia, suicidal ideation and suicide attempts in a sample of 1122 undergraduate students. Anhedonia was rated using the Specific loss of Interest and Pleasure Scale (SLIPS) which is an anhedonia scale centered around the changes in the ability to be interested or take pleasure in primarily social experiences. The results showed that anhedonia was associated with suicidal ideation, even when accounting for depressive symptoms. No significant associations were found with suicide attempts.

A third study [16] using a sample of 404 Persian university students replicated the results of the Winer et al [11] study reporting that anhedonia (rated by the SLIPS) was associated with suicidal ideation and not with suicide attempts, even when depression was controlled for.

One study in a large sample of physicians [21] reported significant associations between anhedonia and suicidal ideations even when depression was controlled for although there was no significant association between anhedonia and suicide attempts. In the same study, when the different items of the anhedonia subscale of the BDI were taken into account and entered concurrently in multiple regression as well as a subscale of the BDI measuring the affective-cognitive component of the BDI, only loss of satisfaction was a significant predictor of suicidal ideation. In other terms, a recent loss of pleasure not limited to social experiences could be a robust predictor of suicidal ideation in medical students.

The results of the present study in medical students strongly suggest that the pattern of association between the different components of anhedonia and suicidal ideation was more similar to that of physicians than that of university students.

The consequences of job dissatisfaction in physicians are, notably, depression and burnout with an increased risk of suicide [20]. Dissatisfaction implies a sense of dislike for, or unhappiness. Major depression and burnout have overlap in physicians in terms of symptoms and the deficiency of the three-dimensional concept of burnout (emotional exhaustion, personal accomplishment and detachment) [32]. Several studies did not support the view that burnout and depression are separate entities [33]. A recent meta-analysis [34] has reported that individual-focused and structural or organizational strategies can result in reductions of the prevalence of burnout in physicians.

Interventional programs have been developed for medical students with a view to increasing mental health service utilization and decreasing suicide risk thus allowing to identify a high proportion of untreated, at risk and potentially suicidal medical students [31].

Several limitations to the present study should be mentioned. Firstly, the results of the study were limited to recent suicidal ideations. In future, the relationships between anhedonia, type D personality and chronic suicidal ideation must also be studied. Secondly, the severity of suicidal ideation was not rated. Thirdly, only self-evaluations were used to measure the different psychometrical variables.

## Conclusion

The current study in medical students suggested a strong link between anhedonia and suicidal ideation that cannot be explained by depression or type D personality.

When all hedonic deficits were taken into account, only recent change of state anhedonia was related with suicidal ideation taking into account the potential effect of depression. When the different components of anhedonia were examined concurrently, only the loss of pleasure measuring dissatisfaction and not the loss of interest in social experiences was associated with suicidal ideation.

Just like physicians, medical students could experience low satisfaction, notably in their jobs or in their medical training. This dimension must be assessed and studied, particularly with a view to offering help in the form of therapeutic and prevention programs.
